# Edible Fungal Polysaccharide–Liposome: Interfacial Interactions, Structure–Function Relationship, and Emerging Application in Oral Delivery and Functional Foods

**DOI:** 10.3390/ijms27157036

**Published:** 2026-08-05

**Authors:** Jiachen Liang, Abdul Mueed, Abdul Basit, Viktoryia Kulikouskaya, Kseniya Hileuskaya, Lijun You

**Affiliations:** 1 School of Food Science and Engineering, South China University of Technology, Guangzhou 510640, China; liangjiachenfst@163.com (J.L.);; 2 State Key Laboratory of Ecological Safety and Sustainable Development in Arid Lands, Xinjiang Institute of Ecology and Geography, Chinese Academy of Sciences, Urumqi 830011, China; 3 Institute of Chemistry of New Materials, National Academy of Sciences of Belarus, 36F. Skaryna Str., 220141 Minsk, Belarusk_hilevskay@mail.ru (K.H.)

**Keywords:** edible fungal polysaccharides, liposomes, β-glucans, oral delivery, fate and biological activity, functional foods, nanocarriers

## Abstract

Liposomes are among the most extensively studied delivery systems owing to their biocompatibility, structural versatility, and ability to improve the stability and bioavailability of bioactive compounds. Meanwhile, edible fungal polysaccharides (EFPs), particularly β-glucans and heteropolysaccharides, have attracted increasing interest because of their antioxidant, immunomodulatory, prebiotic, and health-promoting properties. The integration of EFPs with liposomal systems has emerged as a promising strategy for developing multifunctional nanocarriers with enhanced physicochemical stability and biological performance. However, current research remains fragmented, and the mechanisms by which EFP molecular structures influence liposome assembly, stability, gastrointestinal fate, and delivery efficiency are poorly understood. Moreover, existing reviews primarily focus on liposomes or fungal polysaccharides independently, without systematically addressing their interfacial interactions, structure-function relationships, and translational applications. This review provides a comprehensive and critical overview of EFP liposomes, highlighting the interactions between fungal polysaccharides and lipid bilayers, including hydrogen bonding, electrostatic interactions, hydrophobic association, and surface conjugation. The effects of EFPs on liposomal physicochemical properties, encapsulation performance, membrane stability, gastrointestinal protection, mucoadhesion, cellular uptake, and biological activity are further discussed. Emerging applications in targeted delivery, oral delivery, gut microbiota modulation, gut–brain axis regulation, and functional foods are also critically evaluated. Importantly, this review identifies key research gaps, including the lack of quantitative structure-function relationships, limited understanding of biological transport mechanisms, insufficient investigation of microbiota-mediated effects, and challenges in scalable manufacturing. By integrating glycobiology, nanotechnology, and food science, this review establishes a unified framework for the rational design and future development of EFP-based liposomal delivery systems.

## 1. Introduction

Liposomal drug delivery systems have emerged as one of the most significant advances in pharmaceutical nanotechnology since their introduction in the 1960s. Their unique amphiphilic architecture enables the simultaneous encapsulation of hydrophilic and hydrophobic bioactive compounds, thereby improving physicochemical stability, bioavailability, and controlled release behavior [1,2]. Structurally, liposomes consist of one or more phospholipid bilayers surrounding an aqueous core, allowing the efficient incorporation of diverse therapeutic agents, including small molecules, proteins, nucleic acids, and peptides [3]. Over recent decades, liposomal systems have evolved from conventional vesicles into highly engineered nanoplatforms incorporating PEGylation, surface modification with receptor-specific ligands for active targeting, and stimuli-responsive functionalities [3]. In particular, PEGylation “stealth” liposomes markedly prolong systemic circulation by reducing mononuclear phagocyte system clearance, while stimuli-responsive liposomes enable site-specific drug release in response to endogenous triggers such as pH, redox potential and enzymatic activity or exogenous stimuli including heat, light, ultrasound and magnetic fields [4,5]. These advances have positioned liposomes as versatile delivery platforms with broad applications in pharmaceuticals, nutraceuticals and functional foods.

Concurrently, edible fungal polysaccharides (EFPs) have attracted increasing attention as bioactive macromolecules with extensive therapeutic and nutritional potential. Derived from medicinal and edible fungi such as *Lentinula edodes*, *Ganoderma lucidum*, *Grifola frondosa*, *Hericium erinaceus*, *Pleurotus eryngii*, *Cordyceps militaris*, and *Sanghuangporus sanghuang*, EFPs exhibit a wide range of biological activities, including antioxidant, immunomodulatory, anticancer, anti-inflammatory, antimicrobial, hypoglycemic, hypolipidemic and gut microbiota-regulating effects [6,7,8]. The biological activity of EFPs is strongly influenced by their structural characteristics, including molecular weight, monosaccharide composition, glycosidic linkage patterns, degree of branching, and higher-order conformations. Among these structural features, β-(1→3)-D-glucan backbones with β-(1→6)-linked branches are considered critical determinants of immunomodulatory and antitumor activity [9,10]. Furthermore, triple-helical β-glucan conformations exhibit enhanced affinity toward pattern recognition receptors such as Dectin-1 and Toll-like receptor 4, activating signaling pathways including TLR4/MyD88/NF-*κ*B, MAPK, and NLRP3 inflammasome cascades [11,12]. Through these mechanisms, EFPs modulate macrophage polarization, dendritic cell maturation, natural killer cell activation, and adaptive immune responses, highlighting their potential as promising therapeutic and functional food engineering.

Although substantial progress has independently been achieved in liposome nanotechnology and edible fungal polysaccharide research, studies integrating these two rapidly advancing fields remain fragmented and insufficiently explored. The existing literature primarily focuses either on the pharmacological activities of fungal polysaccharides or on the physicochemical development of liposomal carriers, whereas comprehensive analyses addressing their synergistic integration are still lacking. In particular, the molecular interaction between structurally diverse EFPs and phospholipid bilayers, and their influence on encapsulation efficiency, vesicle stability, release behavior, and preservation of bioactive conformations, remain poorly understood. Furthermore, the translational potential of EFP–liposomal systems across both pharmaceutical and functional food applications has not yet been systematically evaluated. As interest in natural bioactive, nanotechnology-based delivery systems and precision functional foods continues to expand, a critical and integrated review of this emerging interdisciplinary area has become increasingly necessary. Therefore, this review comprehensively summarizes current advances in liposomal delivery systems for EFPs, with particular emphasis on the structural and molecular factors governing encapsulation behavior, stability, targeted delivery, and bioavailability enhancement. This review further evaluates the therapeutic and functional food applications of EFP–liposomal systems, including their immunomodulatory, anticancer, antioxidant, and gastrointestinal health-promoting effects. In addition, current limitations, technological challenges, and future research priorities are critically discussed to provide a unified scientific framework for the rational design and translational development of next-generation liposomal formulations containing edible fungal polysaccharides.

### Literature Search Method

As a narrative review, this article aimed to provide a comprehensive and critical overview of recent advances in edible fungal polysaccharide-functionalized liposomal systems rather than perform a systematic evaluation of the literature. Relevant publications were identified through searches of major scientific databases, including Web of Science, PubMed, Scopus, and ScienceDirect. The literature search primarily covered publications from 2000 to 2026, with earlier landmark studies included as needed to establish fundamental concepts. The search was performed using combinations of keywords including edible fungal polysaccharides, mushroom polysaccharides, β-glucans, liposomes, polysaccharide-functionalized liposomes, nanocarriers, oral delivery, targeted delivery, gut microbiota, and functional foods. Research articles and reviews published in English were prioritized based on their relevance to the molecular interactions, preparation strategies, physicochemical properties, biological mechanisms, and applications of EFP-functionalized liposomal systems. Studies unrelated to edible fungal polysaccharides, non-liposomal delivery systems, conference abstracts, and non-peer-reviewed materials were not considered. The selected literature was critically analyzed to summarize current progress, identify knowledge gaps, and propose future perspectives.

## 2. Structural and Functional Characteristics of EFPs

### 2.1. Effect of Structure on Bioactivity

**Figure 1:**
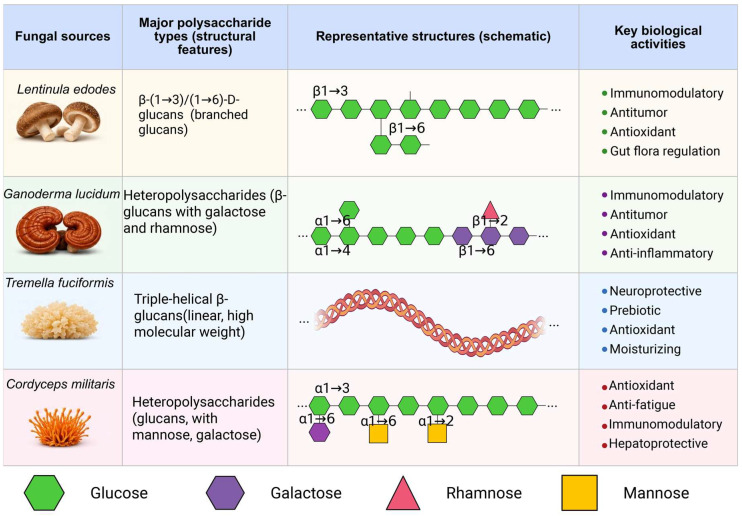
Structural diversity and bioactivity determinants of edible fungal polysaccharides. Note: These structures are “representative simplified structures” rather than universal structures for each fungal species.

EFPs exhibit remarkable chemical diversity arising from substantial variations in monosaccharide composition, glycosidic linkage patterns, degree of branching, molecular weight distribution, and higher-order conformations, all of which collectively govern their physicochemical properties and biological activities [13,14]. Structurally, EFPs encompass glycans, heteroglucans, galactans, mannans, and complex heteropolysaccharides composed predominantly of glucose, galactose, mannose, xylose, fucose, arabinose, rhamnose, and glucuronic acid in highly variable molar ratios depending on fungal species, cultivation conditions, and extraction methodologies [10,15,16]. For example, polysaccharides isolated from *Heimioporus retisporus* contain glucose (49%), mannose (17.78%), galactose (12.93%), xylose (8.59%), and glucuronic acid (10.99%), whereas *Cordyceps cicadae* produces a galactoglucomannan composed of galactose, glucose, and mannose in a 5:1:4 ratio, illustrating the extensive compositional heterogeneity among edible fungi (Figure 1) [17,18]. Among EFPs, β-(1→3)/(1→6)-glucans represent the most extensively investigated class due to their potent immunomodulatory and antitumor properties, where β-(1→3)-linked glucan backbones serve as the primary structural framework and β-(1→6)-linked side chains critically influence molecular flexibility, solubility and receptor accessibility (Table 1). Increasing branching density, particularly branching ratios between 0.2 and 0.33, has been associated with enhanced stabilization of triple-helical conformations and improved recognition by immune receptors [19]. In parallel, molecular weight constitutes another critical determinant of EFP functionality, with native fungal polysaccharides exhibiting extensive heterogeneity ranging from approximately 10 kDa to over 2000 kDa depending on species and purification procedures. High-molecular weight fractions, commonly within 100–800 kDa or higher, generally demonstrate superior immunomodulatory activity owing to enhanced multivalent receptor interactions and preservation of bioactive triple-helical conformations, whereas lower molecular weight fractions enriched with uronic acids or modified functional groups often display improved antioxidant and free radical scavenging activities due to greater molecular mobility and accessibility. For instance, a polysaccharide from *Heimioporus retisporus* exhibited a weight-average molecular weight of 1949 kDa [17], while a β-glucan isolated from *Stropharia rugosoannulata* displayed a molecular weight of 455.6 kDa, highlighting the broad structural variability characteristic of fungal polysaccharides [20]. Furthermore, chemical modifications including sulfation, phosphorylation, carboxymethylation, and acetylation can further modulate electrostatic interactions, receptor affinity, and immunological potency with sulfated derivatives demonstrating significantly enhanced immune cell activation relative to native forms [20].

### 2.2. Triple-Helix Conformation

The triple-helix conformation of β-glucans constitutes a fundamental structural determinant that governs their biological significance, particularly in the context of innate immune recognition and receptor-mediated signaling. β-(1,3)-D-glucan chains spontaneously associate via inter-chain hydrogen bonding to form a stable, rigid, rod-like triple-helical architecture characterized by a hollow core and a repetitive, ordered array of surface epitopes [21,22]. This higher-order organization is not merely a passive physicochemical state but an active immunological signal, as it presents a multivalent scaffold that facilitates high-avidity engagement with Dectin-1 (C-type lectin domain family 7 member A, CLEC7A), the primary pattern recognition receptor (PRR) responsible for β-glucan detection on fungal pathogens [23,24]. Crucially, Dectin-1 signaling is profoundly sensitive to the physical state of its ligand: high molecular weight β-glucans maintaining structured triple-helical conformations induce substantially stronger receptor clustering on the surface of myeloid cells compared to lower molecular weight glucans that adopt disordered random coil configurations [24]. This receptor clustering serves as the prerequisite for recruitment and activation of spleen tyrosine kinase (Syk) via the ITAM-like domain of Dectin-1, initiating the CARD9-Bcl10-MALT1 signalosome and culminating in NF-κB activation and pro-inflammatory cytokine production, including TNF-α, IL-6, and IL-1β [23]. Beyond acute inflammatory responses, the triple-helical structure is particularly potent in inducing “trained immunity” a form of innate immune memory wherein recognition of structured, particulate β-glucans by Dectin-1 triggers metabolic reprogramming toward aerobic glycolysis and accompanying epigenetic modifications (histone methylation and acetylation) at promoters of pro-inflammatory genes, thereby priming myeloid cells for amplified responses to secondary stimulation, with implications for vaccine adjuvancy and cross-protection against heterologous pathogens including SARS-CoV-2 [25,26]. Conversely, soluble or structurally degraded β-glucan forms, which lack the organized triple-helix architecture, generate attenuated Dectin-1 signaling or redirect immune outcomes toward tolerogenic responses, enabling the host to discriminate between intact viable pathogens and inert degradation products [23]. The contribution of the triple-helix conformation to bioactivity is further illustrated by the capacity of structured β-glucans to modulate Th17 cell polarization through dendritic cell activation, direct transient interferon-β expression, and engage complementary receptors such as TLR2 or CR3 in a conformation- and branching-dependent manner [27]. However, the stability of this biologically critical triple-helix structure is notably dependent on solvent environment, temperature and chemical modification. Schizophyllan, a widely studied model β-glucan, undergoes two distinct cooperative order-disorder transitions: a low-temperature transition attributed predominantly to side-chain dynamics and a high-temperature transition involving complete triplex dissociation into single random coils, with the transition temperature (Tr) being solvent-dependent and shiftable to higher values by specific carboxylic acid cosolvents such as acetic acid and citric acid [28]. Furthermore, molecular dynamics simulations of curdlan demonstrated that sulfation at the O_2_ position directly disrupts inter-chain hydrogen bonding networks and significantly destabilizes the triple-helix conformation, whereas substitutions at alternative positions produce variable effects on structural integrity [29], highlighting the exquisite sensitivity of this architecture to site-specific chemical alterations. This indicates that its triple-helix structure is susceptible to disruption during extraction, processing and modification.

### 2.3. Effect of Structure on Absorption

For orally administered EFPs to reach the systemic circulation, they must sequentially overcome the intestinal mucus barrier and the epithelial cell barrier. The mucus layer, mainly composed of mucins, forms the first diffusional and adhesive barrier, where polysaccharide transport is affected by molecular weight, charge, chain flexibility, hydrodynamic size, and interactions with mucin networks [30]. Polysaccharides that are strongly retained by mucus may have limited access to the epithelial surface, whereas those with weaker mucin interactions or smaller hydrodynamic dimensions are more likely to diffuse through the mucus layer. After reaching the epithelium, polysaccharides must further cross the intestinal epithelial barrier through possible pathways such as paracellular transport, transcellular uptake, M-cell-mediated transport, or receptor-mediated endocytosis [31].

Mucus penetration of polysaccharides is determined by their structural features. Molecular weight, charge, and chain flexibility are key determinants. Low-molecular-weight, well-hydrated, and weakly mucoadhesive polysaccharides generally diffuse more readily through the mucin network, whereas high-molecular-weight or strongly adhesive polysaccharides are more likely to be retained by steric obstruction, chain entanglement, electrostatic attraction, hydrogen bonding, or multipoint bridging. Charge plays a central role because mucins are generally negatively charged. Cationic polysaccharides may show enhanced mucoadhesion through electrostatic attraction, whereas anionic or near-neutral polysaccharides may experience weaker adhesion or electrostatic repulsion, which facilitates mucus diffusion. Chain flexibility further modulates this process. Flexible chains may adapt to mucin surfaces and form multiple contacts, thereby enhancing retention, while rigid or less interactive chains may show reduced mucoadhesion and greater mucus mobility [30,32,33].

**Table 1:** Representative structural characteristics of polysaccharide fractions isolated from selected edible fungi.

Fungal Source	Molecular Weight (kDa)	Repeating Unit/Structural Features	Heteropolysaccharide or Glucan	Reference
*Ganoderma lucidum*	16.79 kDa	Major backbone: [→6)-α-D-Galp-(1→]_n_. Partially acetylated residues include [→6)-2-O-Ac-α-D-Galp-(1→]_n_ and [→6)-3-O-Ac-α-D-Galp-(1→]_n_. Terminal side residues include 4-O-Ac-α-L-Fucp-(1→, α-D-Manp-(1→, and α-D-Glcp-(1→.	Heteropolysaccharide	[34]
*Inonotus hispidus*	26.9 kDa	Main-chain motif: [→6)-α/β-D-Glcp-(1→6)-α-D-Galp-(1→6)-3-O-Me-α-D-Galp-(1→6)-α-D-Manp-(1→6)-α-D-Galp-(1→]_n_. A reported side-chain motif contains β-L-Fucp-(1→6)-α-D-Galp-(1→6)-α-D-Glcp-(1→2)-.	Heteropolysaccharide	[35]
*Lactarius hatsudake* Tanaka	LHP-1: 898 kDa; LHP-2: 677 kDa; LHP-5: 4.9 kDa	LHP-1 and LHP-2: [→4)-α-D-Glcp-(1→]_n_. LHP-5: branched β-D-glucan containing β-D-Glcp-(1→6) side residues attached at O-3.	Glucan	[36]
*Pleurotus geesteranus*	20.9 kDa	Major backbones: [→6)-α-D-Galp-(1→]_n_ and [→6)-3-O-Me-α-D-Galp-(1→]_n_. O-2-linked side-chain motif: β-D-Glcp-(1→3)-α-D-Manp-(1→2)-α-D-Galp-(1→, with minor α-L-Fucp residues.	Heteropolysaccharide	[37]
*Pleurotus eryngii*	21.4 kDa	Major backbone: [→6)-α-D-Galp-(1→]_n_ containing β-D-Manp-(1→2) branches. A 3-O-methylated Gal variant was also reported. Minor β-glucan motif: [→6)-β-D-Glcp-(1→]_n_.	Heteropolysaccharide	[38]
*Pleurotus djamor*	161 kDa	Approximate repeat: [→6)-β-D-Glcp-(1→4)-[α-D-Galp-(1→3)]-β-D-Glcp-(1→6)-β-D-Glcp-(1→]_n_.	Heteropolysaccharide	[39]
*Polyporus grammocephalus*	140 kDa	Approximate α-glucan repeat: [→3)-α-D-Glcp-(1→4)-α-D-Glcp-(1→4)-α-D-Glcp-(1→]_n_.	Glucan	[40]
*Grifola frondosa*	2040 kDa	Glucose-dominant motif: [→4)-α-D-Glcp-(1→]_n_, with minor β-D-Glcp-related residues. The reported structure contains both β-D-Glcp-(1→ and α-D-Glcp-(1→4) units.	Glucan-dominant polysaccharide	[41]
*Grifola frondosa*	15.9 kDa	Major backbones: [→6)-α-D-Galp-(1→]_n_ and [→6)-3-O-Me-α-D-Galp-(1→]_n_. Side chains include α-L-Fucp-(1→2)-α-D-Galp-(1→ and α-D-Manp-(1→3)-α-L-Fucp-(1→2)-α-D-Galp-(1→.	Heteropolysaccharide	[42]
*Hypsizygus marmoreus*	21.4 kDa	Major backbone: [→6)-α-D-Galp-(1→]_n_ containing β-D-Manp-(1→2) and/or α-L-Fucp-(1→2) branches	Heteropolysaccharide	[43]
*Pleurotus ostreatus*	22.2 kDa	Major backbone: [→6)-α-D-Galp-(1→]_n_. A 3-O-methylated Gal variant was reported. O-2 branches mainly contain β-D-Manp-(1→2) or α-L-Fucp-(1→2) residues	Heteropolysaccharide	[43]
*Pholiota nameko*	18.9 kDa	Major backbone: [→6)-α-D-Galp-(1→]_n_. Branched variants contain β-D-Manp-(1→2) or α-L-Fucp-(1→2) residues; minor [→6)-3-O-Me-α-D-Galp-(1→]_n_ motifs were also reported.	Heteropolysaccharide	[43]
*Agrocybe cylindracea*	21.0 kDa	Major backbone: [→6)-α-D-Galp-(1→]_n_. O-2 side-chain variants contain β-D-Manp-(1→2), α-L-Fucp-(1→2), or β-D-Manp-(1→?)-α-L-Fucp-(1→2) residues.	Heteropolysaccharide	[43]
*Hericium erinaceus*	22.1 kDa	Major backbone: [→6)-α-D-Galp-(1→]_n_ containing β-D-Manp-(1→2) and/or α-L-Fucp-(1→2) substitutions.	Heteropolysaccharide	[43]
*Meripilus giganteus*	148 kDa	Approximate mixed α/β-glucan motif: [→3)-β-D-Glcp-(1→6)-β-D-Glcp-(1→6)-α-D-Glcp-(1→6)-α-D-Glcp-(1→4)-α-D-Glcp-(1→]_n_. Terminal branches include α-D-Glcp-(1→6)-β-D-Glcp and β-D-Glcp-(1→6)-α-D-Glcp motifs.	Glucan	[44]
*Cordyceps sinensis*	976 kDa	Major branched α-glucan composed of a [→4)-α-D-Glcp-(1→]_n_ backbone containing α-D-Glcp-(1→6) branches.	Glucan	[45]

Abbreviations: Glc, glucose; Gal, galactose; Man, mannose; Fuc, fucose; Ac, *O*-acetyl; Me, *O*-methyl. D and L indicate the absolute configurations of monosaccharides; p denotes the pyranose ring form; and α and β indicate the anomeric configurations. Glycosidic linkages are expressed as α/β-D/L-Sugarp-(1→x), where *x* indicates the substituted carbon atom of the adjacent residue. For example, [→6)-α-D-Galp-(1→]_n_ denotes a repeating chain of 6-linked α-D-galactopyranosyl residues. Square brackets indicate branches or substituents, *n* denotes the number of repeating units, and “?” indicates an unresolved linkage position.

The passive permeability of EFPs across biological membranes is equally and severely impeded by this macromolecular architecture through dual mechanistic barriers. The paracellular pathway permits the intercellular passage of solutes through epithelial tight junctions. Paracellular permeability depends on molecular size and conformation, epithelial type, and tight-junction status. Given the limited evidence regarding the paracellular permeation of polysaccharides, small hydrophilic solutes are used here as a reference, for which transport across intact epithelia is generally limited to molecules with molecular weights below approximately 600–900 Da [46,47]. Transcellular passive diffusion is similarly precluded: governed by the solution-diffusion model whereby permeability is proportional to both the partition coefficient (*K*) and the intra-membrane diffusion coefficient (*P* = *KD*m/*L*) [48], the negligible octanol-water partition coefficients arising from EFP hydrophilicity effectively eliminate any meaningful partitioning into the hydrophobic core of the lipid bilayer [48]. Furthermore, the large hydrodynamic radius conferred by high molecular weight substantially reduces the aqueous diffusion coefficient of EFPs, limiting their capacity to reach and engage membrane surfaces at rates sufficient for meaningful transport [46]. Dectin-1-mediated recognition and internalization constitute an important pathway for the uptake of selected β-glucans, which are among the major components of EFPs [49].

EFPs commonly exhibit molecular weights spanning tens of thousands to several million Daltons, typically neutral or negatively charged [31]. As discussed above, neutral high-molecular-weight polysaccharides are more likely to interact with mucins through hydrogen bonding, chain entanglement, and multipoint association, which can enhance mucus retention and hinder their diffusion across the mucus barrier. Moreover, because of their large hydrodynamic size and strong hydrophilicity, EFPs are unlikely to traverse intestinal epithelial cells by free diffusion or passive transport alone. In the case of certain β-glucans, epithelial uptake may involve receptor-mediated recognition, such as Dectin-1. Furthermore, in terms of solubility, β-glucans in EFPs provide a representative example of how molecular structure constrains aqueous dissolution. Their high molecular weight, together with linear or highly regular chain architecture and a high degree of polymerization, promotes extensive interchain hydrogen bonding, ordered aggregation, and chain entanglement. These strengthened chain–chain interactions reduce polymer hydration and limit chain dispersion in water. Conversely, branching and charged groups can weaken interchain associations and enhance polymer–water interactions, thereby improving solubility. Thus, the poor aqueous solubility of native β-glucans is largely associated with increased structural regularity, high molecular weight, and strong intermolecular association [50].

Compared with most clinically used oral small-molecule drugs, polysaccharide macromolecules generally exhibit markedly lower oral bioavailability. In a dataset of 995 drugs, Kim et al. used F = 50% as the threshold for high versus low human oral bioavailability, with 540 drugs showing F ≥ 50% and 455 showing F < 50%, indicating that many oral drugs can achieve substantial systemic exposure [51]. By contrast, polysaccharide absorption is usually limited by high molecular weight, strong hydrophilicity, and poor transmembrane diffusion. Liao et al. reported that the blood bioavailability of orally administered β-1,3-glucan GFPBW1 and α-1,4-glucan WGE was 18.39% and 4.92%, respectively [49]. Notably, the molecular weights of β-1,3-glucan and α-1,4-glucan are approximately 300 kDa and 100 kDa, respectively [49]. As discussed above, β-glucans with higher molecular weights generally exhibit stronger immunological activity; however, increased molecular weight is also associated with reduced intestinal absorption efficiency. This creates an inherent trade-off between bioactivity and bioavailability, making it difficult to enhance both simultaneously. Therefore, liposome-based encapsulation and delivery, which can improve the intestinal absorption of EFPs, represents a promising strategy for enhancing their overall bioactivity.

## 3. Molecular Interaction Mechanisms Between EFPs and Liposomes

### 3.1. Effects of Lipid Bilayer Composition and Physical State on EFP Interactions

Interaction between β-glucans and phospholipid bilayers arises from a cooperative network of hydrogen bonding, electrostatic interactions, and hydrophobic intercalation, whose relative contributions are governed by membrane composition, state, and polysaccharide conformation, and the lipid compositional landscape of the target membrane [52]. Given the nominal neutrality of β-glucans, their association with phospholipid bilayers is more plausibly dominated by cumulative van der Waals interactions, together with short-range hydrogen bonding. Membrane charge further modulates this process: anionic lipid surfaces enhance electrostatic steering, whereas zwitterionic phosphatidylcholine bilayers promote preferential localization at liquid-ordered/liquid-disordered domain boundaries enriched in packing defects [53]. At sub-nanometre distances, hydrogen bonding dominates interfacial stabilization through multivalent interactions between glucan hydroxyl groups and phospholipid headgroup oxygens [54]. The balance between water-mediated and direct hydrogen bonding is governed by hydration level, with dehydration favoring a water-replacement mechanism that strengthens interfacial adhesion [55]. Cholesterol further reorganizes these interfaces by modulating headgroup hydrogen-bond networks and lipid packing density, thereby increasing accessible sites and, in some cases, enabling partial insertion events not observed in cholesterol-deficient membranes [55]. Hydrophobic intercalation plays a secondary but non-negligible role, arising from transient exposure of lipid packing defects and the amphiphilic character of sugar ring faces. These effects are amplified at domain boundaries, where membrane disorder is maximal [56,57]. Lipid acyl chain saturation strongly regulates all interaction modes: saturated bilayers restrict access through tight packing, whereas unsaturated membranes increase defect density and interfacial plasticity, enhancing adsorption probability [55]. Polymer molecular weight and triple-helix architecture act as cooperative amplifiers of binding. High-molecular-weight β-glucans increase multivalent contact probability and residence time, while triple-helical conformations provide a rigid, hydroxyl-rich scaffold that optimizes spatial complementarity with lipid headgroups [58]. Despite these advances, a quantitative thermodynamic partitioning of electrostatic, hydrogen-bonding, and hydrophobic contributions across defined lipid compositions remains lacking. This limits predictive understanding of β-glucan-membrane interactions and constrains rational design of polysaccharide-functionalized liposomal systems.

### 3.2. Interaction Modes

#### 3.2.1. Hydrogen Bonding

**Figure 2:**
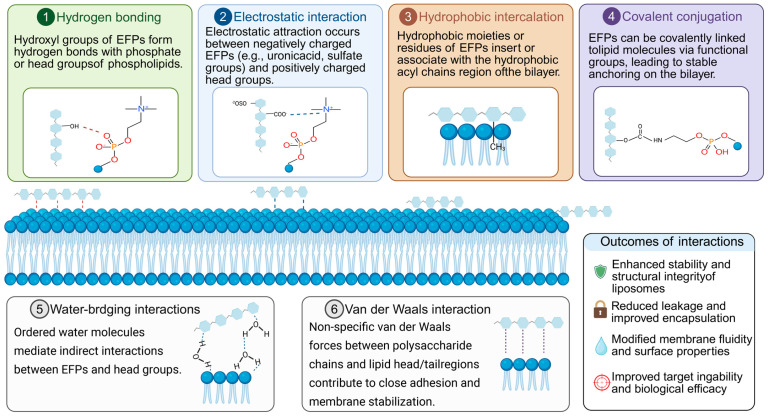
Interfacial interaction mechanisms governing EFP–liposome assembly.

Hydrogen bonding represents one of the most important intermolecular forces governing the association of EFP with liposomal membranes. Unlike electrostatic interactions, which primarily regulate long-range attraction and adsorption, hydrogen bonds provide short-range interfacial stabilization by forming direct contacts between hydroxyl-rich polysaccharide chains and polar phospholipid headgroups (Figure 2) [59]. In fungal polysaccharides, particularly β-(1→3)/(1→6)-glucans, the abundance of hydroxyl groups enables the formation of extensive hydrogen-bonding networks with phosphate, carbonyl, and glycerol moieties located at the membrane-water interface [60]. These interactions contribute substantially to the free energy of adsorption and are often responsible for the stable retention of polysaccharides on liposomal surfaces.

The formation of interfacial hydrogen bonds is strongly influenced by membrane composition and hydration state. In fully hydrated systems, polysaccharide–lipid interactions are frequently mediated by structured water molecules that bridge glucan hydroxyl groups and phospholipid headgroups [61]. As hydration decreases, these water-mediated interactions are progressively replaced by direct hydrogen bonds, resulting in stronger interfacial adhesion and reduced polysaccharide mobility [61]. Cholesterol further modulates this process by reorganizing lipid packing and headgroup orientation, thereby altering the accessibility and distribution of hydrogen-bonding sites within the membrane [61]. Molecular simulation studies suggest that cholesterol-containing bilayers promote more persistent polysaccharide-membrane contacts than cholesterol-free systems owing to enhanced interfacial organization and local packing heterogeneity.

The structural characteristics of EFPs critically determine the extent and stability of hydrogen-bond formation. High-molecular-weight polysaccharides generally establish a greater number of simultaneous contacts with the membrane surface because of their larger interfacial footprint and increased multivalency [62]. Similarly, the triple-helical conformation characteristic of many fungal β-glucans presents a highly ordered array of hydroxyl groups capable of forming cooperative hydrogen-bonding networks with phospholipid headgroups [63]. In contrast, highly branched polysaccharides often exhibit increased hydration and conformational flexibility, which may reduce the density of persistent hydrogen bonds while promoting more dynamic and reversible interactions at the membrane interface [63]. Hydrogen bonding not only governs adsorption but also significantly influences liposomal physicochemical properties. The establishment of polysaccharide-lipid hydrogen-bond networks can reduce membrane permeability, suppress leakage of encapsulated compounds, and enhance vesicle stability during storage and gastrointestinal transit [64]. Furthermore, these interactions contribute to the formation of protective hydration layers around liposomes, improving resistance to aggregation and environmental stresses [65]. In polysaccharide-coated liposomes, hydrogen bonding frequently acts synergistically with electrostatic interactions to stabilize core–shell architectures and maintain coating integrity under physiological conditions [66]. Chemical modification of EFPs can markedly alter hydrogen-bonding behavior. Sulfation, phosphorylation, and carboxymethylation introduce additional hydrogen-bond acceptors while simultaneously modifying hydration characteristics and electrostatic properties [6]. Conversely, acetylation reduces the availability of hydroxyl donors and may weaken direct polysaccharide-lipid hydrogen bonding [67]. These modifications can therefore influence not only coating stability but also membrane fluidity, release kinetics, and biological performance of the resulting liposomal formulations. Despite increasing evidence supporting the importance of hydrogen bonding in polysaccharide–liposome systems, quantitative understanding of its thermodynamic contribution remains limited. Current evidence is limited by the predominant reliance on indirect spectroscopic observations or isolated molecular simulations, whereas few studies have quantitatively determined hydrogen-bond energetics under matched lipid compositions and environmental conditions. Integrated investigations combining calorimetry, vibrational or NMR spectroscopy, membrane-dynamics measurements, and atomistic simulations are therefore required to determine how EFP molecular weight, branching, conformation, and chemical modification affect interfacial hydrogen-bond density and subsequent liposomal performance.

#### 3.2.2. Electrostatic Interactions

Electrostatic interactions constitute one of the principal driving forces governing the adsorption, assembly, and stabilization of EFP liposomes. Unlike hydrogen bonding, which predominantly stabilizes short-range interfacial contacts, electrostatic forces operate over longer distances and therefore play a critical role during the initial stages of polysaccharide-membrane association [59]. The magnitude and direction of these interactions are determined by the surface charge density of the liposome, the ionization state of polysaccharide functional groups, and the physicochemical characteristics of the surrounding medium, including pH and ionic strength.

The electrostatic behavior of EFPs is highly dependent on their chemical composition. Native β-glucans are generally neutral polysaccharides and therefore exhibit limited direct electrostatic interactions with zwitterionic phospholipid bilayers [52]. However, many edible fungal polysaccharides contain uronic acids, phosphate groups, proteins, or sulfate substituents that impart net negative charge under physiological conditions [46]. These anionic moieties can interact strongly with cationic lipid components such as DOTAP or with positively charged polymer-coated liposomes, creating a thermodynamically favorable adsorption process driven by Coulombic attraction [68]. Sulfated polysaccharides are particularly effective because sulfate groups remain ionized across a broad pH range, generating high charge densities that promote strong surface association and enhanced coating efficiency (Figure 2) [69].

Liposome surface charge can be precisely engineered through incorporation of charged phospholipids, enabling controlled modulation of EFP adsorption [70]. Anionic lipids such as DPPG generate negatively charged membrane surfaces that facilitate deposition of cationic biopolymers, whereas cationic lipids such as DOTAP promote association with anionic polysaccharides [68,70]. The resulting polysaccharide layers alter the electrokinetic properties of the vesicles, typically producing measurable shifts in zeta potential and hydrodynamic diameter [70]. These changes reflect successful surface functionalization and frequently correlate with improvements in colloidal stability, storage resistance, and gastrointestinal robustness.

Electrostatic interactions also play a central role in determining the long-term stability of liposomal dispersions. Following adsorption, the magnitude of the resulting surface charge governs interparticle repulsion and aggregation behavior [59]. Liposomal systems exhibiting zeta potential values exceeding approximately ±30 mV generally possess sufficient electrostatic repulsion to resist flocculation and maintain colloidal stability during storage [71]. In contrast, formulations approaching charge neutralization often experience rapid aggregation due to reduced repulsive forces and increased opportunities for particle bridging [71]. Consequently, optimization of the polysaccharide-to-lipid ratio is essential to avoid the instability region associated with charge compensation while maximizing coating efficiency [71]. The influence of electrostatic interactions extends beyond colloidal stabilization to biological performance. Surface charge strongly affects mucus interactions, cellular uptake, protein corona formation, and biodistribution [72]. Positively charged coatings generally exhibit enhanced mucoadhesion owing to electrostatic attraction toward negatively charged mucins, thereby prolonging gastrointestinal residence time and potentially improving oral absorption [73]. Conversely, highly charged surfaces may also promote nonspecific protein adsorption and accelerated clearance following systemic administration [72]. Balancing electrostatic attraction with steric stabilization therefore represents a key design challenge in the development of EFP-coated liposomal systems. The molecular architecture of EFPs additionally affects electrostatic assembly. Polysaccharides possessing higher densities of uronic acids or sulfate groups generally exhibit stronger adsorption onto oppositely charged membranes [69]. Molecular weight and branching architecture further influence adsorption kinetics and layer organization by altering chain flexibility, steric accessibility, and multivalent contact formation [69]. Nevertheless, existing studies have yet to elucidate how EFP charge density, uronic acid and sulfate content, molecular architecture, environmental pH and ionic strength, and the polysaccharide-to-lipid ratio collectively govern adsorption efficiency, zeta potential, colloidal stability, and biological interactions. Systematic factorial investigations employing structurally well-characterized EFP fractions and standardized lipid compositions are therefore required to establish quantitative electrostatic structure-interaction relationships.

#### 3.2.3. Hydrophobic Interaction

Hydrophobic interactions play a more limited but important role in the association of EFP with liposomal membranes. Native fungal polysaccharides, particularly β-(1→3)/(1→6)-glucans, possess dense hydroxyl-group networks that establish extensive hydration shells in aqueous media [60]. The energetic penalty associated with removing these hydration layers renders spontaneous partitioning into the hydrophobic interior of phospholipid bilayers thermodynamically unfavorable. Consequently, most unmodified EFPs remain localized at the membrane-water interface, where they interact primarily with phospholipid headgroups rather than penetrating deeply into the acyl-chain region (Figure 2) [60,74]. Experimental studies employing Langmuir monolayers, NMR spectroscopy, and molecular simulations consistently demonstrate that β-glucans preferentially adsorb onto membrane surfaces while exerting only limited influence on the hydrophobic core of the bilayer [75]. Despite their predominantly hydrophilic nature, subtle hydrophobic contributions can arise from the structural characteristics of polysaccharide chains [76]. Pyranose rings contain relatively nonpolar carbon-hydrogen surfaces that may transiently interact with lipid packing defects exposed at the membrane interface [76]. Such interactions are particularly relevant in membranes exhibiting phase heterogeneity or high cholesterol content, where transient defects and domain boundaries increase local hydrophobic accessibility [76]. Molecular dynamics studies suggest that these weak hydrophobic contacts can cooperate with hydrogen bonding to stabilize polysaccharide adsorption and prolong residence time at the membrane surface [76]. Although individually weak, their cumulative contribution may become significant in high-molecular-weight polysaccharides capable of forming multiple simultaneous contacts [76].

Membrane composition strongly influences the extent of hydrophobic association. Cholesterol-rich bilayers exhibit altered lipid packing, membrane thickness, and interfacial organization, creating regions of increased packing heterogeneity that may facilitate transient insertion of hydrophobic polysaccharide segments or hydrophobically modified derivatives [76]. Similarly, unsaturated phospholipids generate more fluid and disordered membranes with a higher density of packing defects than saturated lipid systems, increasing opportunities for interfacial hydrophobic interactions [77]. These effects are particularly relevant at liquid-ordered/liquid-disordered phase boundaries, which often serve as preferential sites for macromolecular adsorption.

Hydrophobic interactions become substantially more important following chemical modification of EFPs. Conjugation with fatty acids, alkyl chains, cholesterol, phospholipids, or hydrophobic peptides transforms hydrophilic polysaccharides into amphiphilic macromolecules capable of interacting directly with the lipid bilayer core [78]. Such modifications reduce the energetic barrier to membrane partitioning and enable stable anchoring within phospholipid membranes. For example, stearic acid-, cholesterol-, and alkyl-modified polysaccharides exhibit enhanced membrane affinity, improved encapsulation performance, and increased stability of liposomal formulations [78]. In these systems, the hydrophobic moiety acts as a membrane anchor, whereas the polysaccharide chain extends into the aqueous phase to provide steric stabilization, targeting functionality, or bioactivity.

Hydrophobic interactions also influence the physicochemical behavior of liposomal formulations. Anchoring of amphiphilic polysaccharides within the bilayer can alter membrane fluidity, phase-transition behavior, permeability, and mechanical properties [73]. Depending on the degree of insertion, these effects may improve cargo retention and storage stability or, conversely, disrupt membrane organization and increase leakage [73]. Consequently, careful control of hydrophobic modification density and anchor architecture is essential for balancing membrane integration with liposomal integrity. Despite increasing interest in amphiphilic fungal polysaccharide derivatives, the molecular determinants governing hydrophobic association with lipid bilayers remain poorly understood.

#### 3.2.4. Surface Adsorption vs. Covalent Conjugation

Surface functionalization of liposomes with EFP can be achieved through either non-covalent adsorption or covalent conjugation, each offering distinct advantages and limitations. The choice of modification strategy fundamentally influences coating stability, biological performance, circulation behavior, and translational feasibility [70]. Surface adsorption is the simplest and most widely employed approach, relying on electrostatic interactions, hydrogen bonding, and van der Waals forces to associate polysaccharides with the liposomal surface [70]. This strategy preserves the native structure of EFPs and avoids harsh chemical reactions that may compromise bioactivity [70]. Moreover, adsorption can be readily implemented under mild aqueous conditions and is therefore attractive for large-scale production [48]. However, because the interaction is reversible, adsorbed polysaccharides remain susceptible to desorption, competitive displacement by serum proteins, and structural rearrangement under physiological conditions [70]. Consequently, adsorption-based coatings may exhibit reduced long-term stability and less predictable in vivo performance compared with covalently anchored systems [70]. In contrast, covalent conjugation establishes permanent chemical linkages between polysaccharides and lipid components, producing highly stable surface architectures that are resistant to dilution, enzymatic degradation, and protein-mediated displacement [79]. Covalent attachment generally improves coating persistence, prolongs circulation time, and enhances the reproducibility of surface presentation [79]. These attributes are particularly advantageous for targeted delivery applications, where sustained exposure of bioactive polysaccharide motifs is required to maintain receptor recognition and cellular uptake [70]. Various conjugation strategies, including carbodiimide-mediated coupling, thiol-maleimide chemistry, periodate oxidation, and bioorthogonal click reactions, have been successfully employed to generate stable polysaccharide-functionalized liposomes [80]. Among these approaches, click chemistry has attracted particular interest owing to its high specificity, mild reaction conditions, and minimal interference with encapsulated cargo [70].

Despite their superior stability, covalent strategies are not without limitations. Chemical modification may alter polysaccharide conformation, mask biologically active epitopes, or reduce receptor-binding affinity [81]. These concerns are especially relevant for fungal β-glucans, whose immunological activity is strongly influenced by molecular architecture and the accessibility of recognition motifs involved in Dectin-1-mediated signaling [82]. Excessive modification can therefore compromise the very biological functions that motivate the use of EFPs as surface ligands. Furthermore, covalent conjugation introduces additional synthetic complexity, purification requirements, and manufacturing costs, which may limit scalability and regulatory translation.

The relative merits of adsorption and covalent conjugation ultimately depend on the intended application. For functional foods and oral delivery systems, where simplicity, cost-effectiveness, and preservation of native polysaccharide structure are priorities, adsorption-based coatings may be sufficient. In contrast, applications requiring prolonged circulation, active targeting, or precise control of surface composition generally benefit from covalent attachment [81]. An intermediate strategy involves hydrophobic post-insertion of lipid-modified polysaccharides, which combines improved anchoring stability with reduced chemical modification of the polysaccharide backbone [81]. Direct comparisons among adsorption, hydrophobic post-insertion, and covalent conjugation remain limited because these strategies are commonly evaluated using different polysaccharides, lipid compositions, and experimental conditions (Figure 2). Head-to-head studies under matched formulation conditions are needed to determine how attachment chemistry, conjugation site, and surface density affect coating persistence, preservation of bioactive epitopes, receptor recognition, release behavior, and in vivo performance.

### 3.3. Structure Models

EFP–liposome systems can be classified into four structural configurations according to the spatial location of the polysaccharide and its mode of association with the lipid membrane. In core-encapsulated EFP liposomes, EFPs are entrapped within the aqueous core of the vesicles. In surface-adsorbed EFP liposomes, EFPs associate with the outer membrane surface through reversible non-covalent interactions, including hydrogen bonding, electrostatic attraction, and van der Waals forces. In covalently anchored EFP liposomes, EFP chains are chemically linked to functionalized lipids or reactive groups on the liposomal membrane. In lipid-anchored EFP liposomes, hydrophobically modified EFPs are inserted into the lipid bilayer through fatty-acid, alkyl, cholesterol, phospholipid, or other hydrophobic moieties, while the polysaccharide chains extend into the surrounding aqueous phase. These configurations differ in polysaccharide location, coating stability, membrane interaction, preparation requirements, and biological performance. Although surface-adsorbed systems are defined here for conceptual completeness, they are not discussed in detail in this review, which focuses primarily on EFPs encapsulated within the aqueous core or stably anchored to the liposomal membrane through covalent or hydrophobic interactions.

#### 3.3.1. Core Encapsulation

Core encapsulation is the most direct strategy for incorporating EFP into liposomal systems, whereby hydrophilic polysaccharides are entrapped within the aqueous interior of liposomes during vesicle formation [6]. The efficiency of this process is governed by a complex interplay between polysaccharide molecular characteristics, liposome composition, and fabrication conditions [70]. Among these factors, molecular weight is a primary determinant of encapsulation efficiency. Low-molecular-weight polysaccharides generally exhibit higher encapsulation owing to their greater diffusivity and ability to partition into the aqueous compartments formed during liposome assembly [83]. In contrast, high-molecular-weight fungal polysaccharides, including many β-glucans, experience steric restrictions that limit their incorporation into nanoscale vesicles. Molecular conformation further influences encapsulation behavior. Flexible random-coil polysaccharides can adapt to confined aqueous environments more readily than rigid triple-helical structures, which possess larger hydrodynamic dimensions and reduced conformational flexibility [84]. Although rigid conformations may reduce encapsulation efficiency, they often improve post-encapsulation retention by limiting diffusion across the lipid bilayer [1].

Liposome composition also plays a critical role in determining encapsulation performance. Increasing the aqueous volume available within vesicles generally enhances polysaccharide loading, while cholesterol incorporation improves membrane integrity and reduces leakage during storage and gastrointestinal transit [1]. Membrane rigidity, lipid packing density, and bilayer permeability collectively influence the ability of liposomes to retain encapsulated polysaccharides following preparation [56]. Consequently, optimization of lipid composition is often required to balance loading capacity with long-term stability [56]. The preparation method substantially affects encapsulation outcomes. Conventional thin-film hydration remains the most widely used technique because of its simplicity and versatility; however, its encapsulation efficiency for large hydrophilic macromolecules is often limited by the relatively small internal aqueous volume generated during hydration [85]. Reverse-phase evaporation generally provides higher loading efficiencies by producing liposomes with enlarged aqueous cores capable of accommodating high-molecular-weight polysaccharides [85]. More recently, microfluidic approaches have emerged as promising alternatives, offering improved control over particle size distribution, reproducibility, and scalability while minimizing batch-to-batch variability [86]. Nevertheless, systematic comparisons of these techniques for EFP encapsulation remain limited.

Environmental conditions during formulation further influence encapsulation efficiency and retention. Ionic strength, pH, and solution viscosity affect vesicle formation, polysaccharide partitioning, and membrane stability [87]. High-viscosity polysaccharide solutions may hinder homogeneous liposome formation, whereas excessive ionic strength can destabilize liposomal structures and reduce loading efficiency [87]. These effects become increasingly important for high-molecular-weight or highly branched fungal polysaccharides. Accurate evaluation of encapsulation efficiency requires discrimination between truly encapsulated polysaccharides and those merely adsorbed onto the liposome surface. Techniques such as size-exclusion chromatography, ultracentrifugation, and membrane filtration are commonly employed to separate free and surface-associated polysaccharides from entrapped fractions. Failure to distinguish these populations can lead to substantial overestimation of encapsulation performance. Despite considerable progress in liposomal encapsulation technologies, predictive relationships linking EFP molecular architecture, including molecular weight, branching degree, charge density, and triple-helical stability to encapsulation efficiency remain poorly established.

#### 3.3.2. Core–Shell Model

The core–shell model is one of the most widely employed strategies for integrating EFP with liposomal delivery systems. In this architecture, the liposome serves as the inner cargo-containing core, while a polysaccharide layer forms an external shell that modulates interfacial properties, biological interactions, and delivery performance [65]. The resulting hybrid structure combines the encapsulation capability of liposomes with the protective and functional attributes of polysaccharides. The physicochemical characteristics of the shell are primarily determined by the molecular structure of the polysaccharide, including molecular weight, branching degree, charge density, and conformational organization [88]. Upon adsorption or deposition onto the liposomal surface, EFPs generate a hydrated interfacial layer that enhances steric stabilization and reduces vesicle aggregation [89]. This shell can also act as a physical barrier against environmental stresses, including pH fluctuations, enzymatic degradation, and oxidative damage, thereby improving storage stability and gastrointestinal resilience [70].

The biological behavior of core–shell liposomes is strongly influenced by shell composition and thickness [88]. Polysaccharide coatings modify surface charge and hydration, which in turn regulate interactions with mucus, proteins, and cellular membranes [90]. Positively charged or mucoadhesive coatings can prolong gastrointestinal residence by promoting interactions with mucin networks, whereas highly hydrated shells may facilitate mucus penetration by minimizing adhesive interactions [90]. In addition, the shell influences cellular uptake by modulating membrane recognition, endocytic pathways, and particle-cell interactions [86]. Excessively thick coatings, however, may hinder cellular internalization and delay cargo release, highlighting the need to balance protective effects with biological accessibility.

Beyond enhancing physicochemical stability, EFP shells can impart biological functionality. Fungal β-glucans and related polysaccharides possess intrinsic immunomodulatory properties and may interact with pattern recognition receptors such as Dectin-1, complement receptor 3 (CR3), and Toll-like receptors [82]. Consequently, the polysaccharide shell can function not only as a protective coating but also as a bioactive interface that contributes to immune regulation and targeted biological responses [91]. This dual role is particularly attractive for oral immunotherapeutics, vaccine delivery systems, and functional food applications [12,92].

Shell architecture also influences pharmacokinetic behavior. By reducing protein adsorption and limiting premature recognition by the mononuclear phagocyte system, appropriately engineered polysaccharide coatings can prolong circulation time and improve biodistribution [86]. The effectiveness of this protection depends on coating uniformity, shell density, and the persistence of polysaccharide coverage under physiological conditions [86]. Therefore, precise control over shell formation remains a critical design consideration. Despite growing interest in EFP-coated liposomes, quantitative relationships between polysaccharide structure and shell performance remain poorly understood. In particular, how β-glucan molecular weight, branching patterns, triple-helical stability, and chemical modifications influence shell thickness, hydration behavior, cellular interactions, and in vivo fate has not been systematically established.

#### 3.3.3. Anchored Model

The anchored model represents an advanced liposome–polysaccharide architecture in which EFP are covalently or hydrophobically tethered to the lipid bilayer through molecular anchors [70]. Anchoring strategies typically involve covalent conjugation of polysaccharides to functionalized phospholipids or insertion of lipid-modified polysaccharides into preformed bilayers [70]. The resulting constructs exhibit greater resistance to desorption, protein-mediated displacement, and dilution-induced dissociation than non-covalently adsorbed systems [70]. Consequently, anchored liposomes generally display improved colloidal stability, prolonged circulation, and more consistent surface presentation under physiological conditions [70].

A key advantage of the anchored model is the controlled display of bioactive polysaccharide ligands. Anchored presentation can enhance receptor accessibility by projecting polysaccharide chains away from the liposomal surface, thereby facilitating receptor binding and cellular recognition [93]. Such multivalent ligand display has been shown to improve uptake by macrophages, dendritic cells, and other immune cells involved in innate immune responses [93]. Consequently, anchored EFP liposomes have attracted increasing interest for targeted immunotherapy, vaccine delivery, and antifungal applications [93,94].

The performance of anchored systems is strongly influenced by anchor design. Parameters such as linker length, anchor density, spacer flexibility, and conjugation site determine ligand accessibility, membrane stability, and biological activity [95,96]. Flexible spacers, particularly polyethylene glycol (PEG)-based linkers, can improve polysaccharide exposure and reduce steric crowding, whereas excessive ligand density may hinder receptor recognition through conformational restriction or intermolecular interactions [95]. Furthermore, inappropriate conjugation sites may disrupt structural features essential for biological activity, especially in β-glucans whose receptor interactions depend on specific conformational motifs and higher-order organization [97].

Anchored polysaccharides may also influence liposomal pharmacokinetics and biodistribution [96]. Stable ligand presentation can enhance active targeting while reducing premature loss of surface functionality during circulation. In addition, the combination of steric stabilization and receptor-mediated recognition provides opportunities to balance prolonged systemic residence with selective cellular uptake [96]. These characteristics distinguish anchored systems from both conventional coated liposomes and simple adsorption-based formulations [96]. Future studies integrating structural characterization, receptor-binding analysis, and pharmacokinetic evaluation will be essential for optimizing anchored liposomal platforms and realizing the full therapeutic potential of fungal polysaccharide-based nanocarriers.

## 4. Structural Modifications Induced by EFP Incorporation

### 4.1. Physicochemical Properties

#### 4.1.1. Zeta Potential

Zeta potential is a critical physicochemical parameter that governs the stability, interfacial behavior, and biological performance of EFP–liposome systems [98]. The incorporation of EFPs frequently induces substantial changes in liposomal surface charge owing to the presence of ionizable functional groups such as uronic acids, phosphate groups, sulfate substituents, and associated protein residues [6]. Following polysaccharide adsorption or conjugation, a reproducible shift in zeta potential may provide supporting, but not conclusive, evidence of successful surface modification [6]. In particular, anionic EFPs generally decrease the zeta potential of cationic liposomes, whereas positively charged polysaccharide coatings shift surface charge toward more positive values [6]. The magnitude of these changes is influenced by polysaccharide composition, molecular weight, coating density, and liposomal lipid composition [70].

The influence of zeta potential extends beyond physicochemical stability to biological interactions. Surface charge regulates adsorption of biomolecules, interactions with mucus and epithelial barriers, cellular uptake, and biodistribution [6]. Positively charged liposomes generally exhibit enhanced mucoadhesion and cellular association owing to electrostatic attraction toward negatively charged biological surfaces [99]. However, excessive positive charge may also increase nonspecific protein adsorption, complement activation, and rapid clearance by the mononuclear phagocyte system [99]. In contrast, moderately negative or near-neutral surfaces often demonstrate improved systemic compatibility and prolonged circulation [99]. Therefore, the optimal zeta potential depends on the intended delivery route and therapeutic objective.

Environmental factors further modulate zeta potential behavior. Variations in pH alter the ionization state of polysaccharide functional groups, whereas increasing ionic strength compresses the electrical double layer and reduces the magnitude of the measured zeta potential [100]. These effects are particularly relevant for EFP-coated liposomes intended for oral delivery, where exposure to diverse gastrointestinal environments may significantly influence particle stability and surface properties. Future studies should focus on establishing quantitative relationships between EFP molecular architecture including charge density, uronic acid content, sulfation degree, and branching characteristics and zeta potential regulation.

#### 4.1.2. Particle Size and Thickness

Particle size and coating thickness are critical determinants of the physicochemical stability and biological performance of EFP liposomes [66]. The incorporation of EFP generally increases the hydrodynamic diameter of liposomes due to the formation of a surface-associated polysaccharide layer or shell [66]. The extent of this increase depends on polysaccharide molecular weight, chain conformation, coating density, and the mode of attachment [66].

Particle size directly influences colloidal stability, cellular uptake, biodistribution, and gastrointestinal transport [101]. Liposomes within the nanoscale range generally exhibit improved dispersion stability and enhanced interaction with biological barriers, whereas excessively large particles are more prone to sedimentation, aggregation, and rapid clearance [65]. The adsorption of high-molecular-weight or highly branched polysaccharides often produces larger particles due to increased steric volume and hydration, while compact or low-molecular-weight polysaccharides typically generate thinner coatings and smaller hydrodynamic diameters [64].

Coating thickness is equally important because it governs the balance between protection and accessibility. A sufficiently thick polysaccharide layer can improve resistance to aggregation, enzymatic degradation, and environmental stress by providing steric stabilization and a hydrated interfacial barrier [102]. However, excessively thick coatings may hinder cellular interactions, reduce uptake efficiency, and impede the release of encapsulated cargo [102]. Therefore, an optimal shell thickness is required to achieve effective protection without compromising biological performance.

The structural characteristics of EFPs strongly influence shell formation. High-molecular-weight polysaccharides and extended conformations generally produce thicker coatings, whereas branching degree, charge density, and hydration behavior determine shell compactness and uniformity [103]. In addition, coating thickness is affected by liposome surface charge and polysaccharide adsorption efficiency, which regulate the amount of polysaccharide associated with the membrane surface [103].

#### 4.1.3. Surface Hydrophilicity

Surface hydrophilicity is a key determinant of the stability and biological behavior of EFP liposomes [104]. The abundant hydroxyl, carboxyl, and sulfate groups present in EFPs increase the hydration of the liposomal surface, leading to the formation of a hydrated interfacial layer that enhances colloidal stability and reduces particle aggregation [104]. The degree of hydrophilicity is strongly influenced by polysaccharide composition, molecular weight, branching structure, and chemical modifications [102]. Highly hydrated polysaccharides generate thicker hydration layers that provide steric stabilization and improve resistance to environmental stresses, including pH fluctuations, ionic strength changes, and enzymatic degradation [102]. In addition, increased surface hydration can reduce nonspecific interactions with proteins and other biological components, thereby contributing to improved stability in complex physiological environments [102].

Surface hydrophilicity also influences biological interactions. Hydrated polysaccharide layers can enhance mucus penetration, prolong gastrointestinal residence, and modulate cellular uptake depending on the balance between hydration and mucoadhesion [90]. Furthermore, highly hydrophilic surfaces generally exhibit reduced protein adsorption and improved circulation behavior compared with poorly hydrated systems, making surface hydration an important parameter for both oral and systemic delivery applications [90].

The hydrophilic character imparted by EFPs is therefore not only a physicochemical attribute but also a critical factor governing the overall performance of liposomal formulations [105]. Nevertheless, the relationships between EFP molecular architecture, hydration behavior, and biological outcomes remain insufficiently understood. Future studies should establish quantitative correlations between polysaccharide structural features and surface hydration properties to enable the rational design of EFP-coated liposomes with optimized stability and delivery efficiency.

### 4.2. Membrane Dynamics

#### 4.2.1. Phase Transition Temperature

Phase transition temperature (Tm) is a critical parameter that reflects the physical state and stability of liposomal membranes [106]. It represents the temperature at which phospholipid bilayers transition from an ordered gel phase to a more fluid liquid-crystalline phase, thereby influencing membrane permeability, rigidity, and cargo retention [106]. The incorporation of EFPs can alter the phase behavior of liposomal membranes through interactions with phospholipid headgroups and interfacial water layers [104]. Surface-associated polysaccharides often restrict lipid mobility by strengthening interfacial interactions, resulting in increased membrane order and a slight elevation in Tm [56]. Conversely, weakly associated or highly hydrated polysaccharides may disrupt lipid packing and reduce cooperative lipid interactions, leading to a decrease in transition temperature [56]. The magnitude of these effects depends on polysaccharide molecular weight, conformation, coating density, and the strength of polysaccharide–lipid interactions [56]. Changes in Tm have important implications for liposomal performance. An increase in transition temperature generally reflects enhanced membrane rigidity and reduced permeability, which can improve storage stability and minimize premature leakage of encapsulated compounds [106]. In contrast, excessive membrane fluidization may compromise structural integrity and accelerate cargo release [106]. Therefore, modulation of Tm provides valuable insight into the stabilizing or destabilizing effects of EFP incorporation.

The influence of EFPs on membrane phase behavior is also affected by lipid composition. Cholesterol-containing membranes typically exhibit reduced sensitivity to phase transitions because cholesterol broadens the transition region and stabilizes bilayer organization [107]. As a result, the impact of polysaccharide incorporation on Tm is often less pronounced in cholesterol-rich formulations than in pure phospholipid systems [107]. Future studies should establish how structural parameters such as molecular weight, branching degree, and triple-helical conformation influence lipid packing and phase transition characteristics, thereby enabling the rational design of liposomal systems with optimized stability and release properties.

#### 4.2.2. Membrane Fluidity

Membrane fluidity is a fundamental property that influences the stability, permeability, and release behavior of liposomal systems [108]. It reflects the mobility of phospholipid molecules within the bilayer and is highly sensitive to lipid composition, cholesterol content, temperature, and interactions with surface-associated biomacromolecules [108]. The incorporation of EFPs can modulate membrane fluidity through interactions with phospholipid headgroups and the interfacial hydration layer. Polysaccharides that form strong hydrogen-bonding networks with lipid headgroups generally restrict lipid mobility, resulting in decreased membrane fluidity and enhanced bilayer stability [56]. In contrast, weakly associated or highly hydrated polysaccharides may increase interfacial disorder and promote greater lipid mobility [56]. The extent of these effects depends on polysaccharide molecular weight, conformation, coating density, and adsorption strength.

Changes in membrane fluidity have important consequences for liposomal performance. Reduced fluidity is typically associated with improved membrane integrity, enhanced cargo retention, and increased resistance to environmental stress [108]. Conversely, excessive membrane fluidization can increase bilayer permeability and accelerate the release of encapsulated compounds [108]. Therefore, maintaining an appropriate balance between membrane rigidity and fluidity is essential for optimizing liposomal stability and controlled-release properties.

The influence of EFPs on membrane dynamics is further modulated by lipid composition. Cholesterol generally reduces excessive lipid movement and stabilizes bilayer organization, thereby diminishing fluidity changes induced by polysaccharide incorporation [109]. As a result, the impact of EFPs on membrane fluidity is often formulation-dependent and should be considered in conjunction with overall membrane composition. The principal limitation is the absence of quantitative relationships between defined EFP structural parameters and changes in membrane order or phase-transition behavior. Comparative studies combining differential scanning calorimetry, fluorescence anisotropy, membrane-order probes, and structural characterization of the polysaccharides are required to distinguish the effects of molecular weight, branching, conformation, and coating density from those of lipid and cholesterol composition.

#### 4.2.3. Leakage Prevention

Leakage prevention is a critical factor determining the effectiveness of liposomal delivery systems, as premature loss of encapsulated compounds can significantly reduce stability, bioavailability, and therapeutic efficacy [110]. The incorporation of EFPs has been widely recognized as an effective strategy for enhancing cargo retention through stabilization of the liposomal membrane [6]. EFPs reduce leakage primarily by forming a protective interfacial layer around liposomes through hydrogen bonding, electrostatic interactions, and steric stabilization [6]. These interactions strengthen membrane organization, reduce bilayer permeability, and limit the diffusion of encapsulated compounds across the lipid membrane [110]. In addition, the hydrated polysaccharide shell acts as a physical barrier that protects liposomes against environmental stresses such as pH fluctuations, ionic strength changes, and enzymatic degradation [111].

The effectiveness of leakage prevention is influenced by polysaccharide structural characteristics. High-molecular-weight polysaccharides and densely coated shells generally provide greater protection by enhancing steric stabilization and reducing membrane defects [112]. Strong polysaccharide–lipid interactions can further improve membrane integrity by restricting lipid mobility and maintaining bilayer cohesion [112]. Consequently, EFP-coated liposomes frequently exhibit lower leakage rates and improved storage stability compared with uncoated formulations [113].

Membrane composition also plays an important role. Cholesterol-containing bilayers are inherently less permeable and often exhibit synergistic effects with polysaccharide coatings, leading to enhanced cargo retention [114]. The combined optimization of lipid composition and polysaccharide coating therefore represents an effective approach for minimizing leakage while maintaining desirable release characteristics [115]. Future studies should establish quantitative relationships between polysaccharide molecular architecture, coating properties, and leakage behavior to support the rational design of highly stable liposomal delivery systems.

### 4.3. Stability Enhancement

Stability enhancement is one of the primary advantages of incorporating EFPs into liposomal systems. Liposomes are inherently susceptible to aggregation, fusion, oxidation, hydrolysis, and premature cargo leakage during storage and biological application [110]. The presence of EFPs can significantly improve liposomal stability by strengthening interfacial interactions and providing a protective barrier around the lipid membrane [66,104].

The stabilizing effect of EFPs arises from multiple mechanisms. Polysaccharide coatings generate steric and electrostatic repulsion that reduces vesicle aggregation and improves colloidal stability [70]. Simultaneously, hydrogen bonding and other intermolecular interactions between polysaccharides and phospholipid headgroups reinforce membrane organization, reducing structural disruption and permeability [70]. These effects collectively contribute to improved particle integrity and cargo retention.

EFPs also enhance resistance to environmental stresses encountered during processing, storage, and gastrointestinal transit [98]. The hydrated polysaccharide layer can protect liposomes against pH fluctuations, ionic strength variations, enzymatic degradation, and oxidative damage, thereby preserving formulation stability under challenging conditions [70]. Furthermore, high-molecular-weight and highly hydrated polysaccharides often provide greater protection owing to their ability to form robust interfacial networks and steric barriers [70]. The magnitude of stability enhancement depends on both polysaccharide and liposome characteristics, including molecular weight, charge density, coating thickness, lipid composition, and cholesterol content [70]. Appropriate optimization of these parameters is essential, as excessive coating may adversely affect cellular interactions and release behavior despite improving physical stability.

## 5. Fabrication Strategies

### 5.1. Conventional Methods

#### 5.1.1. Thin-Film Hydration

Thin-film hydration is the most widely used method for preparing liposomal formulations owing to its simplicity, versatility, and compatibility with a broad range of bioactive compounds [116]. In this approach, lipids are first dissolved in an organic solvent and dried to form a thin lipid film, which is subsequently hydrated with an aqueous phase containing the target compound [116]. Hydration of the lipid film promotes spontaneous self-assembly of phospholipids into multilamellar vesicles, which can be further processed to obtain liposomes with the desired size and lamellarity [116]. For EFPs, thin-film hydration provides a straightforward strategy for both core encapsulation and surface functionalization [116]. Hydrophilic polysaccharides can be incorporated during the hydration step, while surface-associated polysaccharide coatings may be introduced through post-preparation adsorption [117]. The method is particularly attractive because it requires relatively mild processing conditions, helping preserve the structural integrity and biological activity of fungal polysaccharides [117]. However, encapsulation efficiency is often limited for high-molecular-weight polysaccharides due to the restricted aqueous volume available within liposomal vesicles [118]. In addition, the method typically produces heterogeneous particle populations that require subsequent size-reduction processes, such as sonication or extrusion, to improve uniformity [118]. These limitations may affect reproducibility and scalability, particularly for industrial applications.

#### 5.1.2. Sonication and Extrusion

Sonication reduces vesicle size through the application of ultrasonic energy, which disrupts large multilamellar vesicles and promotes the formation of smaller unilamellar structures [119]. This approach is simple and effective for laboratory-scale preparation; however, excessive sonication may induce lipid oxidation, membrane disruption, or degradation of sensitive bioactive compounds [120]. These concerns are particularly relevant for EFP-loaded liposomes, where preservation of polysaccharide structure and biological activity is essential.

Extrusion is generally preferred when precise size control and narrow particle size distributions are required [119]. In this process, liposomal dispersions are repeatedly passed through membranes with defined pore sizes, producing vesicles with improved uniformity and reproducibility [119]. Compared with sonication, extrusion exerts less stress on the formulation and is therefore more suitable for preparing stable liposomes intended for biological applications [119]. For EFP liposomes, particle size reduction through sonication or extrusion can improve colloidal stability, storage behavior, and biological performance by generating more homogeneous formulations [110]. However, excessive processing may alter polysaccharide coatings, affect surface adsorption, or reduce encapsulation efficiency [110]. Therefore, processing conditions should be carefully optimized to balance particle size control with preservation of liposome integrity and polysaccharide functionality.

#### 5.1.3. Ethanol Injection

Ethanol injection is a simple and scalable method for liposome preparation that relies on the rapid self-assembly of phospholipids following the injection of a lipid-ethanol solution into an aqueous phase [121]. As ethanol diffuses into the surrounding medium, lipid solubility decreases, leading to the spontaneous formation of liposomal vesicles [121]. The method is particularly attractive because it avoids harsh processing conditions and enables the production of relatively small liposomes with narrow size distributions [121]. For EFPs, ethanol injection offers a mild approach for liposome fabrication that can preserve polysaccharide structure and bioactivity [121]. Hydrophilic EFPs may be incorporated in the aqueous phase during liposome formation or introduced subsequently through surface adsorption and coating strategies [113]. Compared with thin-film hydration, ethanol injection often produces smaller and more homogeneous particles while reducing the need for extensive post-processing [122]. However, the encapsulation efficiency of high-molecular-weight polysaccharides is frequently limited due to the rapid vesicle formation process and the relatively small internal aqueous volume of the resulting liposomes. In addition, residual solvent removal and process optimization are important considerations, as solvent composition and injection conditions can significantly influence particle size, encapsulation performance, and formulation stability.

### 5.2. Advanced Technologies

#### 5.2.1. Microfluidics

Microfluidics has emerged as a powerful platform for the controlled fabrication of liposomal delivery systems, offering superior reproducibility, size control, and scalability compared with many conventional methods [123]. The technique relies on the rapid and controlled mixing of lipid and aqueous phases within microscale channels, enabling the spontaneous assembly of liposomes under highly defined conditions [123]. For EFP liposomes, microfluidics provides precise control over particle size, polydispersity, and surface properties, which are critical parameters governing stability and biological performance [123]. The rapid mixing process promotes uniform liposome formation and minimizes batch-to-batch variability, resulting in highly reproducible formulations [123]. In addition, microfluidic platforms can be readily adapted for the incorporation of polysaccharides through either encapsulation or surface functionalization strategies [124]. A major advantage of microfluidics is its ability to generate nanoscale liposomes with narrow size distributions without requiring extensive post-processing [124]. This characteristic is particularly beneficial for EFP-loaded systems, where particle size uniformity can influence colloidal stability, cargo retention, and cellular interactions.

#### 5.2.2. Supercritical Fluids

Supercritical fluid technology has emerged as an attractive alternative for liposome production due to its low solvent requirements, mild operating conditions, and potential for scalable manufacturing [125]. Among the available systems, supercritical carbon dioxide (SC-CO_2_) is the most widely used because of its low toxicity, moderate critical temperature, and ease of removal from the final formulation [125]. In liposome preparation, supercritical fluids facilitate lipid dispersion and vesicle formation while minimizing exposure to harsh organic solvents [125]. This characteristic is particularly advantageous for EFPs, whose structural integrity and bioactivity may be compromised during conventional processing [125]. Compared with traditional methods, supercritical fluid-based techniques can produce liposomes with improved size control, narrow particle size distributions, and reduced solvent residues [125]. The technology also offers advantages for industrial translation, including continuous processing, enhanced reproducibility, and improved environmental sustainability [125]. Furthermore, the mild processing environment may be beneficial for preserving sensitive bioactive compounds encapsulated within EFP liposomes.

#### 5.2.3. Green Approaches

**Figure 3:**
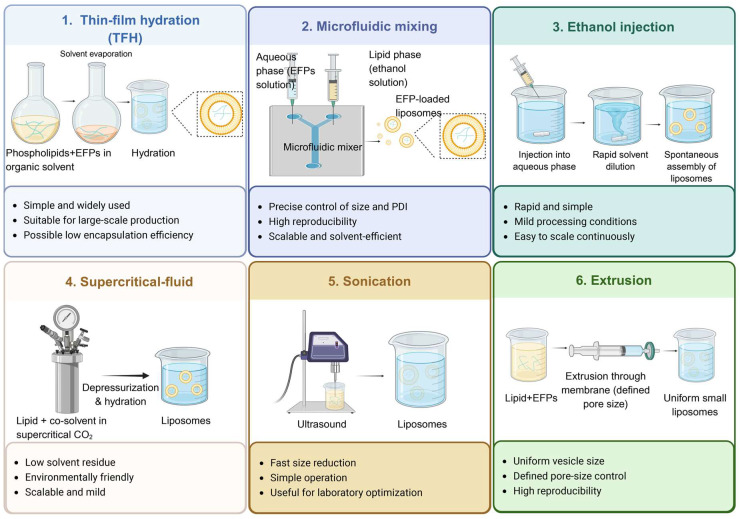
Engineering strategies for EFP-liposomal systems.

Growing environmental and regulatory concerns have stimulated the development of green approaches for liposome production that minimize the use of hazardous solvents, reduce energy consumption, and improve process sustainability (Figure 3) [126]. These strategies are particularly relevant for EFP-based systems, where preservation of bioactivity and food-grade compatibility are essential considerations [126]. Green liposome fabrication typically relies on environmentally benign solvents, solvent-free processes, or low-energy preparation techniques that reduce the environmental footprint of production while maintaining formulation quality [127]. Approaches such as microfluidics, supercritical fluid technologies, and aqueous-based self-assembly methods have attracted increasing attention due to their ability to produce liposomes with controlled physicochemical properties and minimal solvent residues [127]. For EFP liposomes, green processing offers additional advantages by reducing the risk of polysaccharide degradation and preserving structural features that are critical for biological activity [127]. The use of mild processing conditions can improve formulation safety, enhance biocompatibility, and facilitate applications in functional foods, nutraceuticals, and biomedical delivery systems [127].

### 5.3. Encapsulation Metrics

#### 5.3.1. Encapsulation Efficiency

Encapsulation efficiency (EE) is a critical parameter that determines the loading capacity and delivery performance of liposomal formulations. In EFP-based systems, EE reflects the proportion of polysaccharides successfully incorporated within or associated with liposomes relative to the total amount used during formulation [128]. High EE is desirable because it improves formulation efficiency, enhances cargo protection, and reduces material loss during production [128]. The encapsulation efficiency of EFPs is influenced by multiple factors, including polysaccharide molecular weight, conformation, solubility, and liposome composition [128]. Low-molecular-weight polysaccharides generally exhibit higher encapsulation owing to their greater mobility and easier partitioning into the aqueous compartments of liposomes [128]. In contrast, high-molecular-weight or highly branched polysaccharides often show reduced encapsulation because of steric constraints and limited internal aqueous volume [128]. Similarly, rigid conformations such as triple-helical β-glucans may hinder efficient entrapment compared with more flexible polysaccharide structures [57]. Formulation and processing parameters also play important roles. Lipid composition, cholesterol content, lipid-to-polysaccharide ratio, and preparation method can significantly affect EE by influencing vesicle formation and internal aqueous volume [129]. Advanced fabrication techniques, such as microfluidics and reverse-phase evaporation, often provide improved control over encapsulation compared with conventional approaches, although their effectiveness remains dependent on polysaccharide characteristics (Figure 3) [129]. Accurate determination of EE requires effective separation of free polysaccharides from encapsulated or surface-associated fractions [129]. Therefore, interpretation of encapsulation data should consider the analytical methods employed, as incomplete separation may lead to overestimation of loading performance.

#### 5.3.2. Loading Capacity

Loading capacity (LC) is an important parameter that reflects the amount of EFP incorporated into or associated with a liposomal system relative to the total lipid content or carrier mass [130]. The loading capacity of EFP liposomes is governed by both polysaccharide characteristics and formulation design. Polysaccharide molecular weight, conformation, solubility, and charge density influence the extent of encapsulation or surface association [131]. High-molecular-weight polysaccharides may exhibit reduced loading due to steric constraints and limited internal aqueous volume, whereas excessive polysaccharide concentrations can promote aggregation and compromise formulation stability [131]. Consequently, maximizing loading capacity requires balancing payload incorporation with colloidal integrity [131].

Liposome composition also plays a critical role. Factors such as lipid concentration, lipid-to-polysaccharide ratio, membrane composition, and vesicle architecture determine the available loading space and the strength of polysaccharide–liposome interactions [56]. Core-encapsulated systems are often constrained by the aqueous volume of liposomes, whereas surface-coated and anchored architectures can achieve additional loading through interfacial association of polysaccharide chains [56]. From a practical perspective, high loading capacity is desirable because it reduces carrier requirements and improves formulation efficiency [132]. However, excessive loading may alter particle size, surface properties, membrane organization, and long-term stability [132]. Therefore, optimization of loading capacity should consider not only payload content but also its impact on the physicochemical and biological performance of the liposomal system.

## 6. Biological Fate and Mechanisms

### 6.1. Gastrointestinal Stability

#### 6.1.1. Gastric Protection

**Figure 4:**
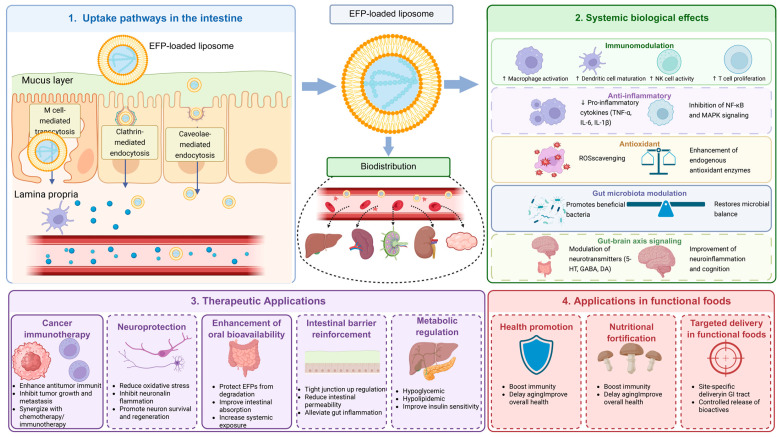
Gastrointestinal fate and biological mechanisms of EFP-loaded liposomes.

Gastric protection is a key requirement for the successful oral delivery of liposomal formulations, as exposure to acidic pH, digestive enzymes, and bile components can destabilize lipid membranes and promote premature cargo release [133]. The incorporation of EFPs provides an effective strategy for enhancing the resistance of liposomes to these harsh gastrointestinal conditions [133]. EFPs improve gastric stability primarily by forming a hydrated protective layer around the liposomal surface (Figure 4) [133]. This polysaccharide coating acts as a physical barrier that limits direct contact between the lipid bilayer and the gastric environment, thereby reducing membrane disruption, vesicle aggregation, and premature leakage of encapsulated compounds [133]. In addition, hydrogen bonding and electrostatic interactions between polysaccharides and phospholipid headgroups contribute to membrane stabilization under acidic conditions [134].

The extent of gastric protection depends on polysaccharide molecular characteristics, including molecular weight, charge density, and coating thickness [135]. High-molecular-weight and highly hydrated polysaccharides generally provide greater protection by enhancing steric stabilization and reducing permeability [135]. Furthermore, polysaccharide-coated liposomes often exhibit improved retention of bioactive compounds during gastric transit, thereby increasing the likelihood of delivery to the intestinal absorption site [70]. Future studies should establish quantitative relationships between EFP structural features and gastrointestinal stability to support the rational design of oral liposomal delivery systems with enhanced protective performance.

#### 6.1.2. Mucoadhesion

Mucoadhesion is an important strategy for improving the oral delivery of liposomal systems by prolonging their residence time at mucosal surfaces and enhancing opportunities for absorption [136]. The incorporation of EFPs can significantly influence mucoadhesive behavior through interactions with the mucus layer covering the gastrointestinal epithelium [133]. The mucoadhesive properties of EFP-coated liposomes primarily arise from hydrogen bonding, electrostatic interactions, and physical chain entanglement between polysaccharide molecules and mucin glycoproteins [90]. These interactions promote adhesion to the mucus layer, reduce rapid luminal clearance, and increase the local concentration of encapsulated bioactive compounds at the absorption site [90]. As a result, EFP liposomes may exhibit enhanced gastrointestinal retention and improved delivery efficiency [133].

The extent of mucoadhesion depends on polysaccharide molecular characteristics, including molecular weight, charge density, branching structure, and hydration behavior [90]. Polysaccharides capable of forming extensive intermolecular interactions with mucin generally display stronger adhesive properties [90]. However, excessive mucoadhesion may restrict diffusion through the mucus barrier, highlighting the need to balance mucus retention with mucus penetration [90]. Beyond improving retention, mucoadhesive coatings can provide an additional protective barrier against gastrointestinal degradation and contribute to sustained release of encapsulated compounds [137]. These combined effects make EFP-coated liposomes attractive candidates for oral delivery applications, particularly for bioactive compounds with limited stability or absorption in the gastrointestinal tract. Despite growing interest in polysaccharide-mediated mucoadhesion, the specific contributions of fungal polysaccharide structure to mucus interactions remain insufficiently understood.

### 6.2. Cellular Uptake

#### 6.2.1. M-Cell Transport

Microfold (M) cells are specialized epithelial cells located within the follicle-associated epithelium of Peyer’s patches and play a central role in the transcytosis of luminal antigens and particulate materials across the intestinal barrier [138]. Owing to their high endocytic activity and relatively thin mucus coverage, M cells represent an important pathway for the oral uptake of nanoscale delivery systems [138]. Available evidence indicates that liposomes can be internalized and transported by intestinal M cells. Ultrastructural studies have visualized intact liposomes within M-cell endocytic vesicles and in vesicular structures between M cells and underlying lymphoid cells, providing morphological evidence consistent with M-cell-mediated transcytosis [139]. Subsequent studies have further shown that surface modification with M-cell-targeting lectins or aptamers can enhance liposomal association, uptake, and transport across epithelial models containing M cells [24,140]. These findings suggest that cargo carried by liposomes can be taken up through the M-cell pathway.

Following uptake, M cells deliver the transported materials to the M-cell pocket and the underlying subepithelial dome, where they can subsequently be recognized by macrophages and dendritic cells. β-Glucans, which are among the major components of EFPs, can interact with pattern recognition receptors expressed on M cells and the underlying antigen-presenting cells, thereby promoting particle uptake and transport across the epithelial barrier [141]. These observations suggest that at least some EFPs encapsulated within liposomes may be transported through the M-cell pathway.

Despite its therapeutic potential, the contribution of M-cell transport to the absorption of EFP liposomes remains insufficiently characterized. Future studies should clarify the relationships between fungal polysaccharide structure, receptor-mediated recognition, and intestinal transcytosis to support the rational design of orally administered liposomal systems with enhanced uptake and bioavailability.

#### 6.2.2. Endocytosis

Endocytosis is a major cellular uptake mechanism that facilitates the internalization of liposomal nanocarriers across intestinal and immune cell barriers [142]. Following interaction with the cell membrane, liposomes can be internalized through multiple pathways, including clathrin-mediated endocytosis, caveolae-mediated endocytosis, macropinocytosis, and receptor-mediated uptake [143]. The dominant pathway depends on particle size, surface charge, lipid composition, and surface functionality [142].

EFPs can significantly influence endocytic uptake by modifying the surface properties of liposomes and promoting interactions with cellular receptors [12]. In particular, β-glucans are recognized by pattern recognition receptors such as Dectin-1, complement receptor 3 (CR3), and selected Toll-like receptors expressed on macrophages, dendritic cells, and intestinal immune cells [82]. These interactions can facilitate receptor-mediated internalization and enhance cellular uptake compared with unmodified liposomes. The efficiency of endocytosis is strongly affected by polysaccharide molecular characteristics [49]. Factors such as molecular weight, branching structure, surface density, and ligand accessibility influence receptor recognition and particle–cell interactions [49]. In addition, nanoscale liposomes with controlled surface properties generally exhibit more efficient uptake than larger or highly aggregated systems [144]. Enhanced endocytosis can improve intracellular delivery, increase bioactive compound absorption, and promote interactions with immune cells, making EFP liposomes attractive platforms for oral delivery, immunomodulation, and vaccine applications [145]. However, the specific uptake mechanisms of fungal polysaccharide-coated liposomes remain insufficiently understood. Future studies should clarify how EFP structure regulates receptor engagement, intracellular trafficking, and biological outcomes to support the rational design of more effective delivery systems.

### 6.3. Biological Effects and Potential Applications

#### 6.3.1. Immunomodulation

Immunomodulation is one of the most distinctive biological properties of EFPs, particularly β-glucans, which are widely recognized for their ability to regulate innate and adaptive immune responses [12]. When incorporated into liposomal systems, EFPs can function not only as structural components but also as bioactive ligands that influence immune cell recognition and activation (Figure 4) [146]. The immunomodulatory activity of EFPs is primarily mediated through interactions with pattern recognition receptors, including Dectin-1, complement receptor 3 (CR3), and Toll-like receptors expressed on macrophages, dendritic cells, neutrophils, and other immune cells [12]. These interactions initiate intracellular signaling pathways that regulate cytokine production, antigen presentation, phagocytosis, and immune-cell activation [12]. Liposomes encapsulating *Ganoderma lucidum* polysaccharides significantly increased serum IgG and IgA antibody production, elevated splenocyte proliferation, and enhanced natural killer cell and macrophage activity compared to free polysaccharides [146]. Liposomal presentation of EFPs can enhance receptor accessibility and prolong biological exposure, thereby improving immunological responses compared with free polysaccharides [146].

The magnitude of immune modulation is closely associated with polysaccharide structural characteristics, including molecular weight, branching degree, glycosidic linkage pattern, and higher-order conformation [12]. In particular, β-(1→3)/(1→6)-glucans with well-preserved conformational structures generally exhibit stronger receptor binding and immunological activity [9]. Consequently, maintaining polysaccharide integrity during liposome fabrication is essential for preserving biological function. Beyond immune stimulation, EFP liposomes may contribute to immune homeostasis by modulating inflammatory responses and improving the delivery of immunologically active compounds [146]. These properties have generated considerable interest in applications such as oral immunotherapy, vaccine delivery, functional foods, and nutraceutical formulations. Despite extensive evidence supporting the immunomodulatory effects of fungal polysaccharides, the influence of liposomal presentation on receptor-specific signaling and downstream immune responses remains incompletely understood. Future studies should establish structure-activity relationships linking EFP molecular architecture, liposomal formulation characteristics, and immunological outcomes to facilitate the rational design of next-generation immunomodulatory delivery systems.

#### 6.3.2. Antioxidant Effects

Antioxidant activity is an important biological function of many EFPs, contributing to their health-promoting and therapeutic potential [147]. Numerous fungal polysaccharides have been reported to attenuate oxidative stress by scavenging reactive oxygen species (ROS), reducing lipid peroxidation, and enhancing endogenous antioxidant defense systems [147]. These properties are particularly relevant in conditions associated with chronic inflammation, metabolic disorders, and tissue damage [147].

The antioxidant capacity of EFPs is closely related to their structural characteristics, including molecular weight, monosaccharide composition, glycosidic linkage patterns, branching degree, and the presence of associated phenolic or protein components [148]. In particular, β-glucans and heteropolysaccharides derived from edible fungi have demonstrated the ability to modulate antioxidant enzymes such as superoxide dismutase, catalase, and glutathione peroxidase, thereby contributing to cellular redox homeostasis [149].

Liposomal incorporation can further enhance the antioxidant effectiveness of EFPs by protecting them from degradation, improving gastrointestinal stability, and increasing bioavailability [2]. In addition, EFP liposomes may provide synergistic antioxidant benefits by combining the intrinsic bioactivity of fungal polysaccharides with the protective effects of the liposomal carrier [2]. These advantages are particularly important for oral delivery systems, where environmental conditions can compromise the stability of free bioactive compounds. Despite growing evidence of antioxidant activity, the mechanisms linking EFP molecular structure to antioxidant performance remain incompletely understood. Future studies should establish structure–activity relationships and evaluate how liposomal formulation influences antioxidant efficacy under physiological conditions.

#### 6.3.3. Gut Microbiota

Modulation of the gut microbiota is an important biological function of EFPs and contributes significantly to their health-promoting effects [133]. As largely non-digestible carbohydrates, many EFPs reach the colon intact, where they serve as fermentable substrates for intestinal microorganisms [133]. This prebiotic activity can selectively stimulate the growth of beneficial bacteria while improving microbial diversity and ecosystem stability.

The microbiota-modulating effects of EFPs are closely associated with their structural characteristics, including molecular weight, monosaccharide composition, branching patterns, and glycosidic linkages [150]. Fermentation of fungal polysaccharides by gut microbes generates short-chain fatty acids (SCFAs), such as acetate, propionate, and butyrate, which contribute to intestinal barrier integrity, immune regulation, and metabolic homeostasis [150]. In addition, EFP supplementation has been associated with increased abundance of beneficial genera, including *Lactobacillus*, *Bifidobacterium*, and *Bacteroides*, while suppressing the growth of potentially harmful microorganisms [151].

Existing evidence indicates that liposomal delivery can substantially enhance the intestinal uptake of edible fungal polysaccharides (EFPs). Wierzbicki et al. reported that, following oral administration of liposome-associated lentinan at a lentinan-equivalent dose of 400 μg, the serum lentinan concentration reached 6422 ± 2287 pg/mL, compared with only 1325 ± 1241 pg/mL after administration of free lentinan [152]. Nevertheless, considering the limited circulating blood volume of mice, these concentrations suggest that only a small fraction of the administered lentinan entered the systemic circulation. This interpretation should be treated cautiously, however, because a serum concentration measured at a single time point does not represent total systemic exposure. Liposomes that are not absorbed intact may be partially disrupted or hydrolyzed in the small intestine, with the extent of phospholipid digestion depending strongly on their lipid composition. For example, approximately 30% of the phospholipids in relatively stable egg-yolk phospholipid liposomes may be hydrolyzed, whereas the hydrolysis rate of soybean phospholipid liposomes can exceed 80% [153]. The resulting lipid digestion products are generally incorporated into mixed micelles and absorbed by the intestinal epithelium. Meanwhile, released EFPs and residual polysaccharide-containing structures may reach the colon, where the polysaccharides can be further degraded and fermented by the gut microbiota, generating beneficial microbial metabolites, such as short-chain fatty acids, that may contribute to host health. Liposomes that escape intestinal uptake and degradation may be metabolized by the colonic microbiota. Gut bacterial phospholipase D can hydrolyze phosphatidylcholine into phosphatidic acid and choline [154], potentially destabilizing liposomes and releasing the encapsulated polysaccharide. Emerging evidence suggests that microbiota-mediated effects contribute substantially to the antioxidant, immunomodulatory, and metabolic benefits associated with fungal polysaccharides [150,151]. However, most existing studies do not distinguish the contributions of the EFP, the liposomal carrier, and the co-encapsulated cargo to microbiota modulation. Moreover, causal relationships among microbial community changes, metabolite production, intestinal barrier function, and host responses have rarely been established. Controlled formulation comparisons integrated with metagenomic, metabolomic, and host physiological analyses are therefore required.

#### 6.3.4. Potential of Gut–Brain Axis Modulation

The gut–brain axis is a bidirectional communication network linking the gastrointestinal tract, gut microbiota, immune system, and central nervous system [155]. Increasing evidence suggests that EFPs can influence this axis by modulating gut microbial composition, microbial metabolites, and immune signaling pathways, thereby contributing to neurological and metabolic health [133,155].

As fermentable polysaccharides, EFPs can promote the growth of beneficial gut microorganisms and stimulate the production of short-chain fatty acids (SCFAs), including acetate, propionate, and butyrate [133]. These metabolites play important roles in maintaining intestinal barrier integrity, regulating neuroinflammation, and influencing neural signaling through immune, endocrine, and vagal pathways [133,150]. In addition, EFP-mediated modulation of the gut microbiota may reduce systemic inflammation and oxidative stress, both of which are closely associated with neurological dysfunction [133].

Liposomal delivery offers opportunities to enhance the gut–brain axis-related benefits of EFPs by improving gastrointestinal stability and controlled intestinal release [156]. Furthermore, EFP liposomes may serve as dual-function systems that combine microbiota modulation with the delivery of neuroprotective or bioactive compounds, potentially strengthening gut–brain communication and associated health outcomes [156]. Despite growing interest in microbiota–brain interactions, the specific mechanisms through which EFP-loaded liposomes influence the gut–brain axis remain largely unexplored. Future studies should clarify the relationships among polysaccharide structure, microbial metabolism, metabolite production, and neurological responses to support the development of targeted liposomal systems for gut–brain axis modulation.

## 7. Food Applications

### 7.1. Effect of the Food Matrix on Liposome

Effect of ionic strength on liposomes. Ionic strength is an important determinant of liposomal colloidal stability. Increasing electrolyte concentration screens surface charges and compresses the electrical double layer, thereby weakening electrostatic repulsion between vesicles and potentially promoting aggregation, fusion, and leakage of encapsulated compounds. These effects are particularly pronounced for charge-stabilized liposomes, whereas multivalent ions may additionally bind or bridge charged phospholipid headgroups. Nevertheless, sensitivity to ionic strength depends strongly on membrane composition, surface charge, and bilayer rigidity. Crommelin demonstrated that changes in NaCl concentration altered the ζ-potential and physical stability of charged phosphatidylcholine-cholesterol liposomes, although experimentally observed stability was not always fully predicted by classical electrostatic theory [157]. Liposomes prepared from milk fat globule membrane phospholipids exhibited thicker and less permeable bilayers and greater resistance to ionic and environmental stresses than those prepared from soybean phospholipids [158]. Moreover, surface coating with whey protein can reduce salt-induced osmotic effects and improve liposomal stability in food-like media [159].

Protein–liposome interactions. Proteins interact with liposomes through electrostatic attraction, hydrophobic interactions, hydrogen bonding, and van der Waals forces, resulting in surface adsorption or partial insertion into the phospholipid bilayer. The interaction is governed by protein concentration, conformation and net charge, as well as liposome surface charge, lipid composition, membrane fluidity, pH, and ionic strength. Whey protein–liposome complexation has been shown to induce rearrangement of both protein and lipid groups and to modify protein surface hydrophobicity and interfacial properties [160]. Under acidic conditions, partially unfolded whey proteins may insert into the membrane and form a protective coating that increases bilayer rigidity, decreases permeability, and improves resistance to salts, sugars, and low pH [159]. However, the stabilizing effect is protein-dependent: whey protein predominantly forms a surface layer, whereas lysozyme may penetrate more deeply into the membrane [161]. Excessive protein adsorption, charge neutralization, or protein-mediated bridging can instead promote vesicle aggregation. In biological fluids, protein adsorption also produces a protein corona, whose composition and extent depend strongly on liposome surface charge and bilayer fluidity and can substantially alter liposomal surface properties and biological fate [162].

Other food-matrix constituents, including sugars, lipid droplets, and organic acids, may also interact with liposomes. Sugars such as glucose, sucrose, and lactose alter water activity, viscosity, and osmotic pressure. An osmotic imbalance across the phospholipid bilayer can cause vesicle shrinkage or swelling, membrane deformation, transient pore formation, and cargo leakage, with the extent of damage depending strongly on membrane lipid composition [163,164]. By contrast, when present at suitable concentrations on both sides of the membrane, sugars may reduce vesicle collision and provide osmotic stabilization. Lipid droplets can interact directly with liposomes at the oil-water interface. Liposomes may adsorb onto the droplet surface and act as Pickering stabilizers, forming an interfacial barrier that suppresses droplet aggregation and coalescence; however, competition with proteins and low-molecular-weight emulsifiers, as well as lipid exchange between the droplet interface and liposomal bilayer, may alter liposome integrity and cargo distribution [165]. Organic acids, including citric, lactic, and acetic acids, affect liposomes mainly through acidification and changes in the ionization of phospholipid headgroups and surface-coating materials. A low pH may reduce electrostatic repulsion, promote aggregation and leakage, and accelerate acid-catalysed phospholipid hydrolysis. For example, greater leakage was observed at pH 3 than at approximately pH 4, although the co-encapsulation of citric and ascorbic acids provided substantial protection of ascorbic acid in apple juice and fermented milk [166]. Liposome stability in acidic beverages can be further improved by incorporating membrane stabilizers or depositing protective biopolymer layers, as demonstrated for low-methoxyl-pectin-coated liposomes in orange juice [167]. Overall, the effects of a food matrix result from the combined actions of osmotic pressure, pH, interfacial adsorption, molecular exchange, and interactions among sugars, acids, lipids, proteins, and phospholipid membranes.

### 7.2. Effect of Processing on Liposomes

The physicochemical stability of liposomes is highly sensitive to processing and storage conditions, which may alter membrane organization, vesicle integrity, and the retention of encapsulated compounds. Thermal treatment increases phospholipid bilayer fluidity, particularly near or above the lipid phase-transition temperature, thereby promoting membrane defects, vesicle aggregation or fusion, and leakage of hydrophilic encapsulated compounds. For example, autoclaving at 121 °C for 15 min caused pronounced leakage of water-soluble compounds and phospholipid hydrolysis, even when no significant changes in liposome size were observed [168]. Nevertheless, the effects of heat processing depend strongly on lipid composition, cholesterol content, pH, and the properties of the encapsulated compound. Appropriately formulated liposomes have retained acceptable structural and oxidative stability during juice pasteurization, indicating that their resistance to processing can be improved through rational membrane design [169]. Freezing may concentrate liposomes and solutes within the unfrozen phase, generating osmotic stress, membrane deformation, vesicle fusion, and leakage of encapsulated compounds during freezing and thawing [170]. Drying, particularly freeze-drying, may further disrupt vesicle organization by removing membrane-associated water, inducing phospholipid phase transitions, and promoting aggregation or fusion after reconstitution. However, cryo- and lyoprotectants such as sucrose and trehalose can reduce membrane damage and improve the retention of vesicle size and encapsulated compounds during freezing, drying, and subsequent rehydration [171].

Fan et al. found that storage temperature markedly affected the encapsulation stability of phosphorylated *Auricularia cornea* polysaccharide-loaded liposomes (P-ACPL). After 28 days, the encapsulation efficiencies of P-ACPL stored at −20, 4, and 25 °C were 75.80%, 83.36%, and 60.48%, respectively. A similar trend was observed for the corresponding liposomal gel (P-ACPLG), which retained encapsulation efficiencies of 65.63%, 75.32%, and 58.41% after 60 days at −20, 4, and 25 °C, respectively. These results demonstrate that refrigeration at 4 °C was more effective than freezing or room-temperature storage in preserving the encapsulation stability of the polysaccharide-loaded liposomes [66]. Overall, the stability of liposomal systems during processing and storage depends on the combined effects of temperature, membrane composition, hydration state, and protective additives, emphasizing the need for formulation-specific optimization to minimize membrane disruption and cargo leakage.

### 7.3. Challenges

Practical translation will further require evidence extending beyond laboratory-scale formulation. Priority areas include long-term shelf-life evaluation in representative food matrices, batch-to-batch reproducibility, determination of an effective dietary dose, sensory assessment, consumer acceptance, cost analysis, and comparison with simpler food-grade delivery systems. Scale-up studies should monitor whether increases in production volume alter vesicle size distribution, coating uniformity, encapsulation efficiency, oxidation, and release behavior. Regulatory and safety requirements must also be considered, particularly when formulations contain a substantial nanoscale particle fraction. Regulatory assessment should consider the fungal species and source material, extraction and purification procedures, batch consistency, food-grade status of phospholipids and other excipients, residual solvents, contaminants, allergenicity, particle-size distribution, and safety at the proposed intake level. Given the substantial research gaps that remain, future studies should first address these unresolved scientific, technological, and safety issues, thereby establishing the evidence base required to support the development of regulatory frameworks for EFP–liposome systems. Thus, the successful translation of EFP–liposome systems into functional foods will require scalable and reproducible manufacturing, stability in realistic food matrices, acceptable sensory properties, demonstrated safety and regulatory compliance, and substantiated nutritional benefits at practical dietary doses [172,173,174,175].

## 8. Conclusions and Future Perspectives

EFP liposomes have emerged as a promising delivery platform that integrates the structural advantages of liposomal nanocarriers with the intrinsic bioactivities of fungal polysaccharides. Current evidence indicates that EFPs can enhance liposomal stability, gastrointestinal protection, cargo retention, and biological performance. However, most available studies remain at the laboratory or proof-of-concept stage, and their practical translation is constrained by several challenges. The structural and batch variability of EFPs limits formulation reproducibility and the establishment of quantitative structure-function relationships. In addition, non-standardized preparation and characterization methods, insufficient stability evaluation in complex food matrices, and changes induced by processing, storage, and gastrointestinal digestion hinder reliable performance assessment. Scale-up may further affect particle size, coating uniformity, encapsulation efficiency, and production cost, while sensory quality, long-term safety, regulatory requirements, and consumer acceptance remain insufficiently evaluated. Future research should therefore prioritize structurally characterized EFPs, standardized analytical methods, validation in representative food matrices, and scalable food-grade manufacturing. Addressing these scientific and translational barriers will be essential for advancing EFP liposomes from experimental formulations to reproducible functional food and nutraceutical products.

## Data Availability

No new data were created or analyzed in this study. Data sharing is not applicable to this article.
